# AgeViva Modulates Inflammatory Responses, Autophagy, and Mitochondrial Homeostasis in BV-2 Cells and *C. elegans*

**DOI:** 10.3390/metabo16090625

**Published:** 2026-08-28

**Authors:** Federica Armeli, Emily Schifano, Beatrice Mengoni, Arianna Montanari, Martina Menin, Laura Pompa, Maria Luisa Crudeli, Thomas Lenz, Trevor Archer, Daniela Uccelletti, Rita Businaro

**Affiliations:** 1 Department of Medico-Surgical Sciences and Biotechnologies, Sapienza University of Rome, 04100 Latina, Italy; federica.armeli@uniroma1.it (F.A.); beatrice.mengoni@uniroma1.it (B.M.); menin_martina@yahoo.it (M.M.); marialuisa.crudeli@uniroma1.it (M.L.C.); 2 Department for Well-Being, Health and Environmental Sustainability—BeSSA, Sapienza University of Rome, Via Della Fontanelle, 02100 Rieti, Italy; 3 Department of Biology and Biotechnologies “C. Darwin”, Sapienza University of Rome, 00185 Rome, Italy; ari.montanari@uniroma1.it (A.M.); laura.pompa@uniroma1.it (L.P.); daniela.uccelletti@uniroma1.it (D.U.); 4 Milmed Unico AB, 11139 Stockholm, Sweden; thomas.lenz@milmed.se (T.L.); trevorcsarcher49@gmail.com (T.A.); 5 Based Metabolomics Laboratory (NMLab), Sapienza University of Rome, 00185 Rome, Italy

**Keywords:** oxidative stress, neuroinflammation, autophagy, mitochondrial dysfunction, reactive oxygen species, ageing, neurodegeneration, Nrf2 signalling, microglia, probiotics

## Abstract

**Background**: Oxidative stress, neuroinflammation, impaired autophagy, and mitochondrial dysfunction are major contributors to ageing and neurodegenerative diseases. This study investigated whether AgeViva could counteract these processes by modulating redox balance, inflammation, autophagy, and mitochondrial function. **Methods**: The effects of AgeViva were evaluated using two complementary experimental models: lipopolysaccharide (LPS)-stimulated BV2 microglial cells and *Caenorhabditis elegans* (*C. elegans*) nematodes. In BV2 cells, the expression of inflammatory, autophagy-related, and antioxidant response genes, including NRF2, SOD, and GPX, was evaluated by RT-qPCR, while cell viability was assessed by Trypan Blue exclusion assay. In *C. elegans*, lifespan, healthspan parameters, ROS accumulation, mitochondrial integrity, membrane potential, and the expression of stress-response, longevity, and autophagy-related genes were analyzed following AgeViva supplementation. **Results**: In LPS-stimulated BV2 cells, AgeViva significantly reduced the expression of mRNA the pro-inflammatory cytokines Interleukin-1 beta (IL-1β) and Tumor Necrosis Factor alpha (TNF-α) while increasing Interleukin-10 (IL-10) levels, AgeViva also induced changes in autophagy-related transcripts, such as modulation of microtubule-associated protein 1a/1b-Light Chain (LC3) and Sequestosome 1 (p62) expression, activated antioxidant-related gene expression, increasing the expression of Superoxide Dismutase 1(SOD1) and Glutathione Peroxidase (GPX). In *C. elegans*, AgeViva supplementation extended lifespan and improved healthspan parameters, including locomotor activity and pharyngeal pumping. Treated nematodes showed reduced cytosolic and mitochondrial ROS accumulation, preservation of mitochondrial network integrity, and maintenance of mitochondrial-associated fluorescence, reflecting mitochondrial content and/or membrane potential during ageing. Molecular analyses revealed modulation of key pathways involved in stress resistance and longevity, including Insulin-like Growth Factor 1 (Insulin/IGF-1) signaling Dauer Formation-2 and 16 (DAF-2/DAF-16), Skinhead-1 (SKN-1/Nrf2) signaling, and autophagy-related genes, like Ligating (*lgg-1*), Autophagy-Related-7 (*atg-7*), Autophagy Related-18 (*atg-18*), uncoordinated-51 *(unc-51*), and ectopic p-granules autophagy protein 5 (*epg-5*). **Conclusions**: AgeViva promotes healthy ageing by modulating oxidative stress, inflammation, autophagy, and mitochondrial homeostasis.

## 1. Introduction

Oxidative stress and autophagy are two closely interconnected cellular mechanisms that play crucial roles in the development and progression of several neurodegenerative diseases, including Alzheimer’s disease (AD), Parkinson’s disease (PD), and Amyotrophic Lateral Sclerosis (ALS). The accumulation of ROS and the malfunctioning of autophagy systems may contribute significantly to the neuronal damage and cellular dysfunction observed in these disease conditions [1]. Although several studies have explored these processes separately, the interaction between oxidative stress and autophagy remains an evolving area of research, with important implications for the development of therapeutic strategies. Chronic inflammation is a key factor in the development of many neurodegenerative disorders, wherein an abnormal inflammatory response contributes to neuronal damage and disease progression [2]. Microglia, which are the innate immune cells of the central nervous system, are activated during inflammation, and the release of pro-inflammatory cytokines such as TNF-α, IL-1β, and Interleukin-6 (IL-6) amplifies the inflammatory response and neuronal damage. Under conditions of oxidative stress, the activation of inflammatory signalling pathways, such as the nuclear factor kappa-light-chain-enhancer of activated b cells (NF-κB) pathway, may be enhanced, creating a feedback loop that contributes to the neurodegenerative evolution [3]. Oxidative stress occurs when the production of ROS exceeds the cell’s antioxidant defence capacity, causing damage to proteins, lipids, and deoxyribonucleic acid (DNA). This cellular damage can trigger a series of cellular responses, including the activation of autophagy, a process by which the cell degrades and recycles damaged or unnecessary cytoplasmic components, including organelles, misfolded proteins, and pathogens, to maintain homeostasis. These processes occur through the formation of a double membrane, called an autophagosome, which engulfs the material to be degraded and fuses it with the lysosome for digestion. The efficiency of autophagy is regulated by a series of key proteins, including LC3 and p62 [4]. LC3 is a membrane protein that binds to the autophagosome membrane during the process of autophagosome formation. An increase in LC3 is associated with the formation of autophagosomes and, consequently, with enhanced autophagic activity. On the other hand, p62 is a protein that acts as a “scaffold” for the selection and recruitment of damaged proteins or protein aggregates, facilitating their recognition and degradation through autophagy [5]. However, if not properly regulated, autophagy can also contribute to the pathogenesis of neurodegenerative diseases by promoting the incomplete clearance of misfolded proteins and aggregates, which are characteristic features of many neurodegenerative diseases [1]. Oxidative stress is a pathophysiological phenomenon caused by the excessive accumulation of ROS, which include free radicals, such as the superoxide anion (O_2_^−^), hydrogen peroxide (H_2_O_2_), and the hydroxyl radical (OH·) [6]. These highly reactive compounds damage proteins, lipids, and nucleic acids, compromising cellular integrity and triggering inflammatory responses. The O_2_^−^, in particular, is one of the most common ROS and is produced primarily by mitochondria during cellular respiration [7]. In the context of neurodegenerative diseases, the superoxide anion plays a key role in neuronal damage and the activation of cell death pathways. Since the superoxide anion is unstable and can be rapidly converted into other forms of ROS, such as H_2_O_2_, controlling its production is essential for the maintenance of cellular homeostasis. Mitochondria, of central importance for energy production and ROS management, are also the primary targets of oxidative stress, resulting in mitochondrial dysfunction that further fuels the cycle of cellular damage in neurodegenerative disorders [8,9]. In this context, BV2 microglia cells and *C. elegans* are widely used experimental models for investigating the mechanisms underlying oxidative stress and autophagy in neurodegenerative diseases [5]. BV2 microglial cells, which serve as a useful model for studying inflammatory responses and oxidative stress in the brain, were used to examine the effect of pro-inflammatory stimuli, such as LPS, on the regulation of these processes. On the other hand, *C. elegans*, a simple yet powerful animal model, offers a translational approach for studying the physiology of oxidative stress and autophagy in a multicellular organism, with unique advantages in terms of genetic accessibility and direct observation of physiological changes in vivo [5]. The present study investigates the potential role of AgeViva, a dietary supplement containing Milmed, zinc, calcium D-pantothenate, vitamin D3, and magnesium, in modulating cellular pathways associated with oxidative stress, inflammation, autophagy, and mitochondrial homeostasis. Increasing evidence suggests that gut microbiota-derived interventions and nutritional compounds may influence systemic and cellular responses involved in ageing and neurodegeneration by regulating redox balance, inflammatory signalling, and proteostasis mechanisms.

AgeViva contains Milmed yeast, a treated yeast-derived component previously investigated for its potential effects on oxidative stress and inflammatory responses in experimental models. However, the biological effects of AgeViva on microglial inflammatory responses, oxidative stress regulation, autophagy, and organismal ageing-related phenotypes remain to be fully characterized. To address this gap, we evaluated the effects of AgeViva supplementation in two complementary experimental models: BV-2 microglial cells and *C. elegans*. BV-2 cells were used to investigate the effects of AgeViva on inflammatory signalling, oxidative stress-related responses, and autophagy-associated pathways under cellular stress conditions. *C. elegans* was employed as an established model organism to assess ageing-related phenotypes, mitochondrial homeostasis, oxidative stress responses, and healthspan-associated parameters [10].

We hypothesized that AgeViva supplementation modulates stress-response pathways involved in inflammation, redox regulation, and autophagy, thereby promoting cellular homeostasis and improving ageing-related physiological outcomes. The primary outcomes of this study were the evaluation of (i) pro- and anti-inflammatory and antioxidant gene expression responses in BV-2 cells; (ii) autophagy-related molecular changes; (iii) oxidative stress and mitochondrial parameters; and (iv) healthspan-associated phenotypes and ageing-related molecular responses in *C. elegans*.

## 2. Materials and Methods

### 2.1. AgeViva

AgeViva is a dietary supplement formulated as hydroxypropyl methylcellulose (HPMC) capsules containing 400 mg per capsule of Milmed yeast obtained from *Saccharomyces cerevisiae* DSM 33148 (Milmed yeast) after exposure to millimetre electromagnetic wavelengths (range: 1 GHz to 300 GHz) [11]. The preparation is supplemented with 1.5 mg zinc (as anhydrous zinc gluconate), 0.9 mg calcium D-pantothenate (equivalent to 0.9 mg pantothenic acid), and 1 μg vitamin D3 (cholecalciferol; 40 IU). The formulation also contains magnesium salts of fatty acids as an excipient, with a total capsule weight of 420 mg. In the present study, to discriminate the specific effects of the electromagnetic-treated live yeast from those of the full commercial formulation containing micronutrients, two preparation methods were employed: (i) ‘AgeViva’ (liquid form/live cells), consisting of re-cultured viable *S. cerevisiae* DSM 33148 cells isolated on solid agar medium, and (ii) ‘AgeViva Powder’, representing the complete capsule content (containing dried Milmed yeast, zinc gluconate, calcium D-pantothenate, vitamin D3, and magnesium stearate) directly suspended in the culture medium.

### 2.2. Cell Culture

The murine microglial cell line BV-2, generously provided by Dr. Mangino (Sapienza University of Rome, Latina, Italy), was cultured in Dulbecco’s Modified Eagle’s Medium (DMEM; Sigma-Aldrich, St. Louis, MO, USA) supplemented with 10% fetal bovine serum (FBS; Sigma-Aldrich, St. Louis, MO, USA), 1% penicillin-streptomycin, L-glutamine, sodium pyruvate, and non-essential amino acids (Sigma-Aldrich, St. Louis, MO, USA). Cells were maintained at 37 °C in a humidified atmosphere with 5% CO_2_.

Cell Line Details: https://www.cellosaurus.org/CVCL_0182 (accessed on 1 June 2026)

- Database/Catalogue ID: CVCL_0182;
- Species: Mouse (Mus musculus);
- Origin: Derived from primary microglia (C57BL/6 strain) and immortalized with v-raf/v-myc oncogenes (J2 retrovirus);
- GEO Accession GSE103156: Global transcriptome analysis of BV-2 cells treated with LPS to evaluate anti-inflammatory agents.

### 2.3. Trypan Blue Assay

A Trypan Blue Assay was performed to assess BV-2 microglial cell viability following treatment. BV-2 cells were seeded in 48-well plates at a density of 3 × 10^4^ cells/well in 500 μL of complete culture medium. AgeViva treatments were administered either as a liquid suspension of re-cultured yeast (AgeViva liquid form) or as raw capsule powder directly suspended in the medium (AgeViva powder). Prior to each treatment, a viability assay (CFU determination and hemocytometer counting) was conducted to quantify the number of live yeast cells per treatment dose. Treatments were added at concentrations equivalent to 5 × 10^2^, 10^3^, and 10^4^ viable yeast cells/well. Following a 24 h incubation at 37 °C in a humidified atmosphere containing 5% CO_2_, culture supernatants were removed, and cell monolayers were washed three times with sterile phosphate-buffered saline (PBS) to thoroughly eliminate non-adherent yeast cells, extracellular debris, and insoluble powder particles. Attached BV-2 cells were then detached using Trypsin-Ethylene diaminetetraacetic Acid (EDTA) (AU-L0940-100, Aurogene, Rome, Italy). Cell suspensions were mixed in a 1:1 ratio with Trypan Blue solution (Corning, Glendale, AZ, USA) and counted using a Bürker chamber under phase-contrast microscopy. BV-2 microglial cells (15–20 μm) were visually distinguished and gated from residual yeast cells 3–5 μm) based on cell size and membrane morphology. Cell viability was determined based on Trypan Blue exclusion, where viable BV-2 cells remained colourless and non-viable cells incorporated the dye.

### 2.4. Real-Time Quantitative PCR Analysis for BV2 Cells

Cells were seeded in 6-well plates (cell density of 106 cells/well) and incubated at 37 °C with 10^3^ AgeViva liquid or powder for 45 min, after 45 min BV-2 cells were treated with LPS (LPS strain 0111:B4, Sigma Aldrich, St. Louis, MO, USA) 1 ng/mL (4h) and compared to control. The total Ribonucleic Acid (RNA) was extracted from both the BV2 control and treated cells using the miRNeasy Micro Kit (Qiagen, Hilden, Germany) and quantified by a NanoDrop One/OneC (Thermo Fisher Scientific, Waltham, MA, USA). The Complementary DNA (cDNA) was synthesized using a high-throughput reverse transcription kit. Real-Time Quantitative Polymerase Chain Reaction (qPCR) was performed for each sample in triplicate on a CFX Opus 96 Real-Time PCR System (Bio-Rad Laboratories, Inc., Hercules, CA, USA) using SsoAdvanced™ Universal SYBR^®^ Green Supermix (Bio-Rad Laboratories, Inc., Hercules, CA, USA). Primers for real-time PCR amplification were designed through the UCSC Genome Browser https://genome.ucsc.edu/ (accessed on 1 November 2022) (Table 1). Analysis of the real-time PCR data was performed using the comparative threshold cycle (CT) method. The target quantity, normalized against the endogenous β-actin reference primer (∆CT) and against the untreated control calibrator (∆∆CT), was calculated by the 2^−∆∆CT^ equation. Experiments were performed in triplicate (both biological and technical replicates).

### 2.5. C. elegans Strains and Growth Conditions

The *C. elegans* strains used in this study were the wild-type N2 Bristol strain and the transgenic strain SJ4103 [zcIs14 (*myo-3*::GFP(mit))]. Worms were maintained under standard laboratory conditions on Nematode Growth Medium (NGM) agar plates. Age-synchronized populations were obtained as previously described [12]. NGM plates (3.5 cm diameter) were supplemented with 60 μL of untreated *S. cerevisiae* yeast or AgeViva suspension prepared in sterile double-distilled water at a final concentration of 50 mg/mL and uniformly distributed to generate the food lawn.

### 2.6. Lifespan and Healthspan Assays

Lifespan analyses were carried out at 16 °C using synchronized worm populations. Briefly, for synchronization, gravid adult hermaphrodites were transferred onto fresh NGM plates and allowed to lay eggs for 8 h. After the egg-laying period, adult worms were removed, and the deposited embryos were allowed to develop under the experimental conditions. Synchronized populations of age-matched worms were subsequently used for all downstream assays. Animals were transferred daily onto freshly prepared NGM plates containing untreated *S. cerevisiae* or AgeViva lawns to avoid progeny contamination. Worms were scored dead when they failed to respond to gentle mechanical stimulation with a platinum wire. Animals that died because of non-age-related causes (e.g., vulval rupture, desiccation) were treated as censored and not included in the statistical analysis. At least 80 nematodes were analyzed for each experimental condition. Healthspan was evaluated by measuring pharyngeal pumping and locomotor activity in 1-day-old and 10-day-old adult worms continuously exposed to untreated or AgeViva yeasts from hatching. Measurements were performed over a 30 s interval under a Leica SZ30 stereomicroscope (Leica Microsystems, Wetzlar, Germany). All assays were conducted through three independent biological experiments with three technical replicates per condition.

### 2.7. Analysis of Mitochondrial Morphology in myo-3::GFP Worms

Mitochondrial morphology was assessed in transgenic SJ4103 worms expressing the mitochondrial reporter Myosin-3 (*myo-3*): Green Fluorescent Protein (GFP). Synchronized worms grown on untreated yeast or AgeViva-supplemented plates were analyzed at days 5, 10, and 15 of adulthood. Animals were immobilized using sodium azide (20 mmol L^−1^; Sigma-Aldrich, St. Louis, MO, USA) and examined with a Zeiss Axiovert 25 fluorescence microscope (Zeiss, Oberkochen, Germany). For each experimental group, 15 worms were evaluated, and all experiments were performed through three independent biological experiments with three technical replicates per condition.

### 2.8. Determination of Cytosolic and Mitochondrial Reactive Oxygen Species (ROS) and Mitochondrial Functionality

To evaluate oxidative status, fluorescence signals associated with oxidative stress were measured in 1-day-old and 10-day-old adult worms previously grown on AgeViva or untreated *S. cerevisiae* yeast. Oxidative status-related fluorescence was assessed using the oxidation-sensitive probe (H_2_DCF-DA; Sigma-Aldrich, St. Louis, MO, USA). Worms were collected, washed in M9 buffer, and incubated with the fluorescent dye for 1 h at 20 °C in the dark under gentle agitation. For oxidative challenge experiments, worms were exposed to H_2_O_2_ at 50 µM for 1 h prior to fluorescence analysis. Fluorescence intensity was quantified using a Tecan Infinite 200 microplate reader (Tecan Group Ltd., Männedorf, Switzerland) with excitation and emission wavelengths set at 485 and 520 nm, respectively. Mitochondrial-associated oxidative fluorescence was evaluated in N2 worms at day 1 and day 10 of adulthood using MitoSOX™ Red (Invitrogen, Thermo Fisher Scientific, Waltham, MA, USA). Worms were incubated for 1 h with 5 μM dye, and fluorescence signals were subsequently analyzed by fluorescence microscopy. Mitochondrial-associated fluorescence was evaluated in 1- and 10-day-old wild-type adult worms using MitoTracker^®^ Red CMXRos (Thermo Fisher Scientific, Schwerte, Germany). Animals were incubated with 5 μM MitoTracker^®^ Red CMXRos for 1 h, washed, immobilized on 3% agarose pads with 20 mM sodium azide, and imaged under a Zeiss Axiovert 25 microscope. MitoTracker^®^ Red CMXRos fluorescence was used to assess mitochondrial staining intensity, which may reflect changes in mitochondrial content and/or membrane potential. Fluorescence intensity was quantified as mean fluorescence intensity (MFI) using ImageJ software v.1.54. Background fluorescence was subtracted prior to analysis, and measurements were performed on images acquired under identical imaging conditions. For ROS evaluation, three independent biological experiments were performed, with 3 technical replicates per experimental condition.

### 2.9. Quantitative Real-Time PCR Analysis for C. elegans

Total RNA was isolated from approximately 200 synchronized 1-day-old and 10-day-old adult nematodes for each condition. Gene expression analysis was performed by Quantitative Real-Time PCR (qRT-PCR) as previously described in [13]. Briefly, RNA was reverse-transcribed into cDNA and amplified using gene-specific primers listed in Table 2. Relative gene expression levels were normalized to the selected housekeeping genes (*act-1* for oxidative stress genes and *pmp-3* for autophagy genes) and calculated using the comparative 2^−ΔΔCt^ method. All experiments were performed in triplicate: each biological replicate consisted of an independently synchronized nematode population from which RNA extraction, cDNA synthesis, and subsequent analysis were performed. qPCR reactions were carried out using technical replicates to ensure analytical reproducibility.

### 2.10. Statistical Analysis

For all the employed techniques, statistical analyses were performed using GraphPad Prism 9.0 software (GraphPad Software Inc., La Jolla, CA, USA). Differences between groups were evaluated using Student’s *t*-test or two-way.

ANOVA followed by Bonferroni’s post hoc test, as appropriate. Lifespan data were analyzed using Kaplan–Meier survival curves and compared using the log-rank (Mantel–Cox) test. Statistical significance was defined as *p* < 0.05 and indicated as follows: * *p* < 0.05, ** *p* < 0.01, *** *p* < 0.001, and **** *p* < 0.0001. All experiments were independently repeated at least three times. Data are expressed as mean ± Standard Deviation (SD) or ±Standard Error of the Mean (SEM).

## 3. Results

### 3.1. AgeViva Promotes Cell Viability and Population Expansion

**Figure 1:**
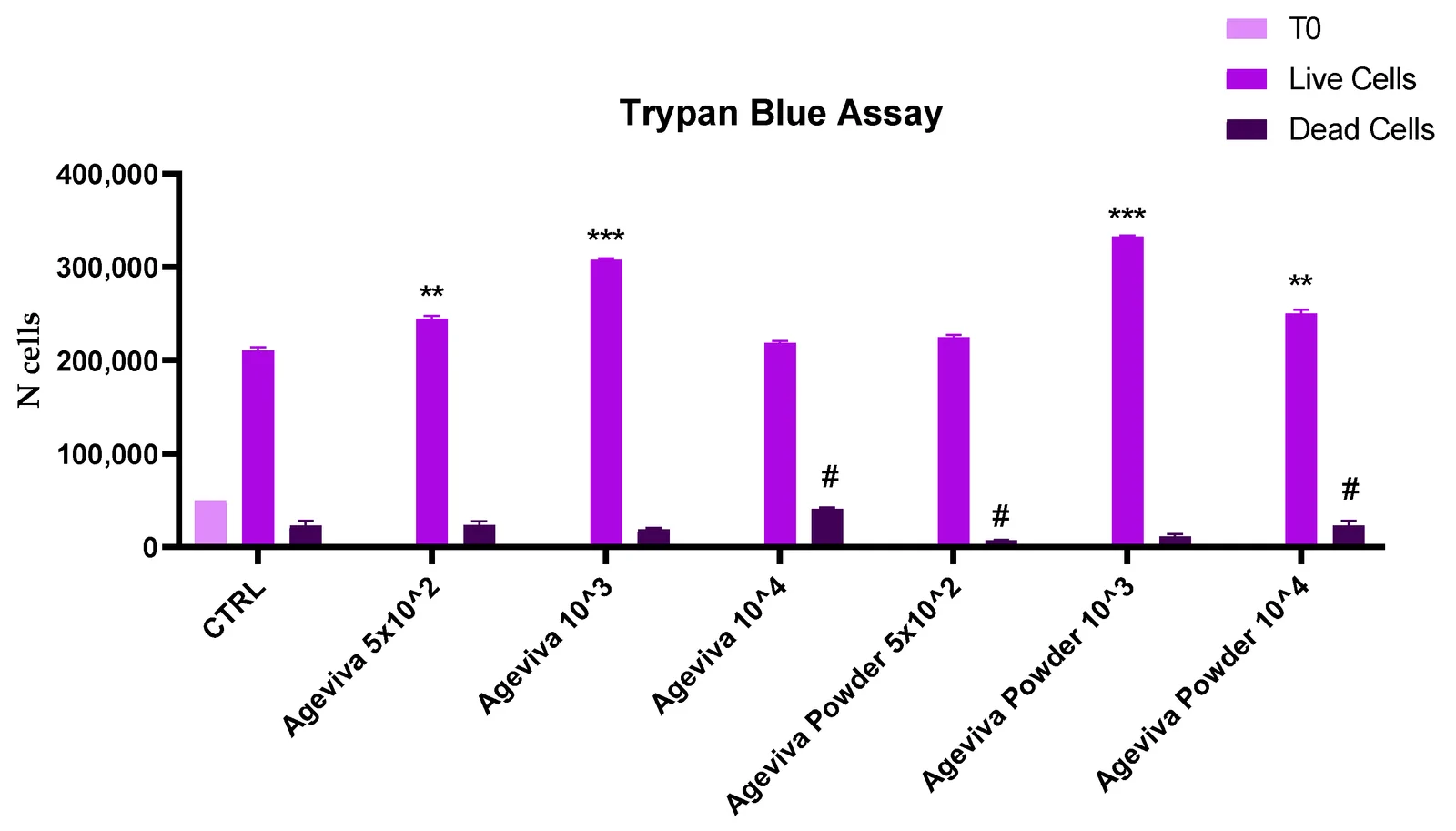
Trypan Blue Assay for AgeViva cytotoxicity. Data are reported as mean ± SD from three independent experiments; statistical analysis was performed using the unpaired Student’s *t*-test. * vs. Live cells; # vs. Dead cells; ** *p* < 0.01; *** *p* < 0.001; # *p* < 0.05.

To evaluate the effect of AgeViva and AgeViva Powder on BV-2 microglial dynamics, a Trypan Blue exclusion assay was performed after 24 h of exposure, starting from an initial seeding density of 3 × 10^4^ cells/well (T0) (Figure 1). In untreated Control (CTRL) cultures, BV-2 cells exhibited robust proliferation, corresponding to a 90.1% cell viability and a 7.0-fold population expansion relative to T0. Treatment with liquid AgeViva at 5 × 10^2^ and 10^3^ cells/well induced a statistically significant, dose-dependent increase in live cell counts (*p* < 0.01 and *p* < 0.001 vs. CTRL). This corresponded to a 10.2-fold population expansion and an elevated viability rate of 95.8%, suggesting an enhancement of microglial proliferation/cell density expansion under non-cytotoxic conditions. Similarly, AgeViva Powder at 10^3^ cells/well yielded 11.1-fold expansion and 97.1% viability; *p* < 0.001. Conversely, at higher exposure levels (AgeViva 10^4^ and AgeViva Powder 5 × 10^2^ and 10^4^), while total live cell counts remained comparable to or slightly above control levels, a modest increase in dead cells was recorded (*p* < 0.05), reducing overall viability to approximately 83.5% for AgeViva 10^4^. These results indicate that while BV-2 cells maintain high baseline viability across all tested conditions (83%), intermediate concentrations (10^3^ cells/well) optimal for promoting cell expansion without inducing membrane compromise were selected for subsequent mechanistic experiments. These findings demonstrate that AgeViva enhances cell viability and supports microglial cell proliferation under basal conditions, rather than merely altering the live/dead cell ratio.

### 3.2. AgeViva Anti-Inflammatory Effect

**Figure 2:**
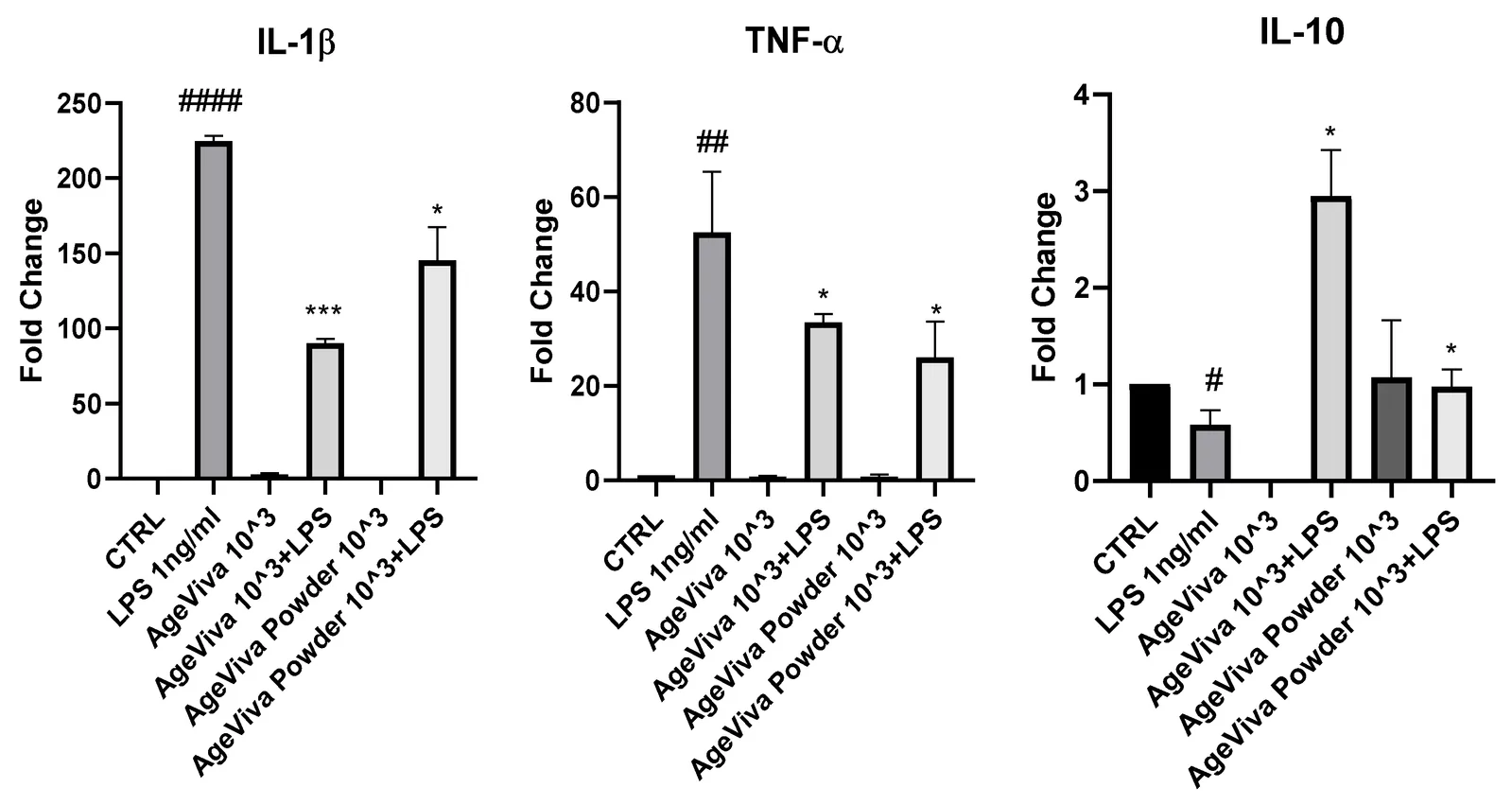
IL-1β, TNF-α and IL-10 mRNA expression in BV-2 cells after AgeViva pre-treatment (45 min) and LPS stimulation (1 ng/mL for 4 h). Data are shown as the mean ± SD from three independent experiments performed in triplicate. Expression profiles were determined using the 2^−ΔΔCT^ method. vs. CTRL # *p* < 0.05, ## *p* < 0.01, #### *p* < 0.0001; vs. LPS * *p* < 0.05, *** *p* < 0.001.

LPS stimulation induces a marked increase in IL-1β mRNA (*p* < 0.0001), confirming the inflammatory activation of BV2 cells. AgeViva (10^3^) in the presence of LPS significantly reduces IL-1β expression (*p* < 0.001), indicating an anti-inflammatory effect, whereas AgeViva Powder (10^3^) shows a lower decrease (*p* < 0.05). A similar trend is observed for TNF-α mRNA: LPS induces a significant increase in TNF-α mRNA (*p* < 0.01) and pre-treatment with AgeViva and AgeViva Powder (10^3^) significantly reduces TNF-α mRNA following LPS stimulation (*p* < 0.05). LPS alone reduces IL-10 mRNA expression (*p* < 0.05). However, pre-treatment with AgeViva (10^3^) results in a marked increase in IL-10 Messenger Ribonucleic Acid (mRNA) (*p* < 0.05), suggesting the activation of anti-inflammatory mechanisms. AgeViva Powder (10^3^) induces a lower increase. Overall, the data show that in LPS-stimulated BV2 cells, AgeViva: (i) significantly reduces the expression of the pro-inflammatory IL-1β and TNF-α mRNAs; and (ii) increases the expression of the anti-inflammatory cytokine IL-10 mRNA (Figure 2).

### 3.3. Modulation of Microglial Inflammatory Transcript Profiles by AgeViva

**Figure 3:**
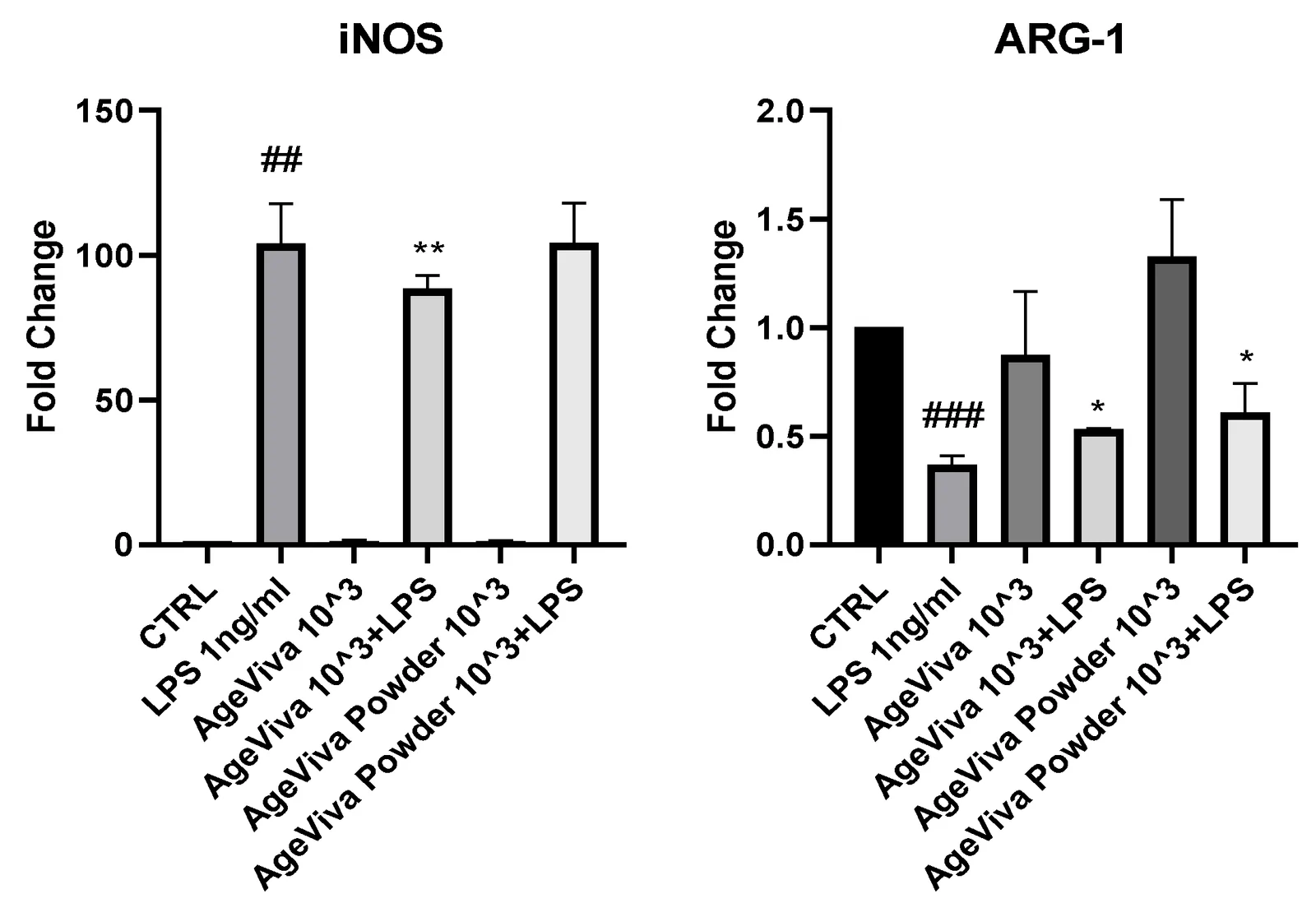
RT-qPCR analysis of iNOS (**left**) and ARG-1 (**right**) mRNA levels in BV2 microglial cells. The cells were treated with LPS (1 ng/mL) in the presence or absence of AgeViva and AgeViva Powder (10^3^). Data are expressed as fold change relative to the control (CTRL). Symbols indicate statistical significance: ## *p* < 0.01; vs. CTRL; ### *p* < 0.001 vs. CTRL; * *p* < 0.05; ** *p* < 0.01 vs. LPS.

To evaluate the modulatory potential of AgeViva and its powder formulation on microglial polarization, the expression of Inducible Nitric Oxide Synthase (iNOS) mRNA (M1 pro-inflammatory marker) and Arginase-1(ARG-1) (M2 anti-inflammatory marker) was analyzed by RT-qPCR in BV2 cells (Figure 3). Exposure of BV2 cells to LPS (1 ng/mL) induced a marked and significant increase in iNOS mRNA compared to the control (CTRL), confirming the pro-inflammatory activation (*p* < 0.01). Pretreatment with AgeViva (10^3^) in the presence of LPS led to a significant reduction in iNOS mRNA compared to the stimulation with LPS only (*p* < 0.01), suggesting anti-inflammatory activity. In contrast, the AgeViva Powder formulation (10^3^) administered in combination with LPS did not show a significant reduction in iNOS mRNA, compared to the treatment with LPS alone. Concurrently, ARG-1 mRNA expression was assessed to determine the activity on inflammation resolution. Treatment with LPS caused a drastic and significant reduction in ARG-1 mRNA expression compared to the control (*p* < 0.0001). Treatment with AgeViva (10^3^) did not significantly reverse the LPS-induced decline in ARG-1 mRNA. Treatment with AgeViva and AgeViva Powder alone did not significantly alter baseline ARG-1 mRNA levels, although the powder showed a trend toward an increase compared to the control. The addition of LPS in the presence of AgeViva or AgeViva powder showed a low recovery of ARG-1 mRNA compared to the addition of LPS only.

### 3.4. Changes in Autophagy-Related Transcript Expression in BV2 Cells

**Figure 4:**
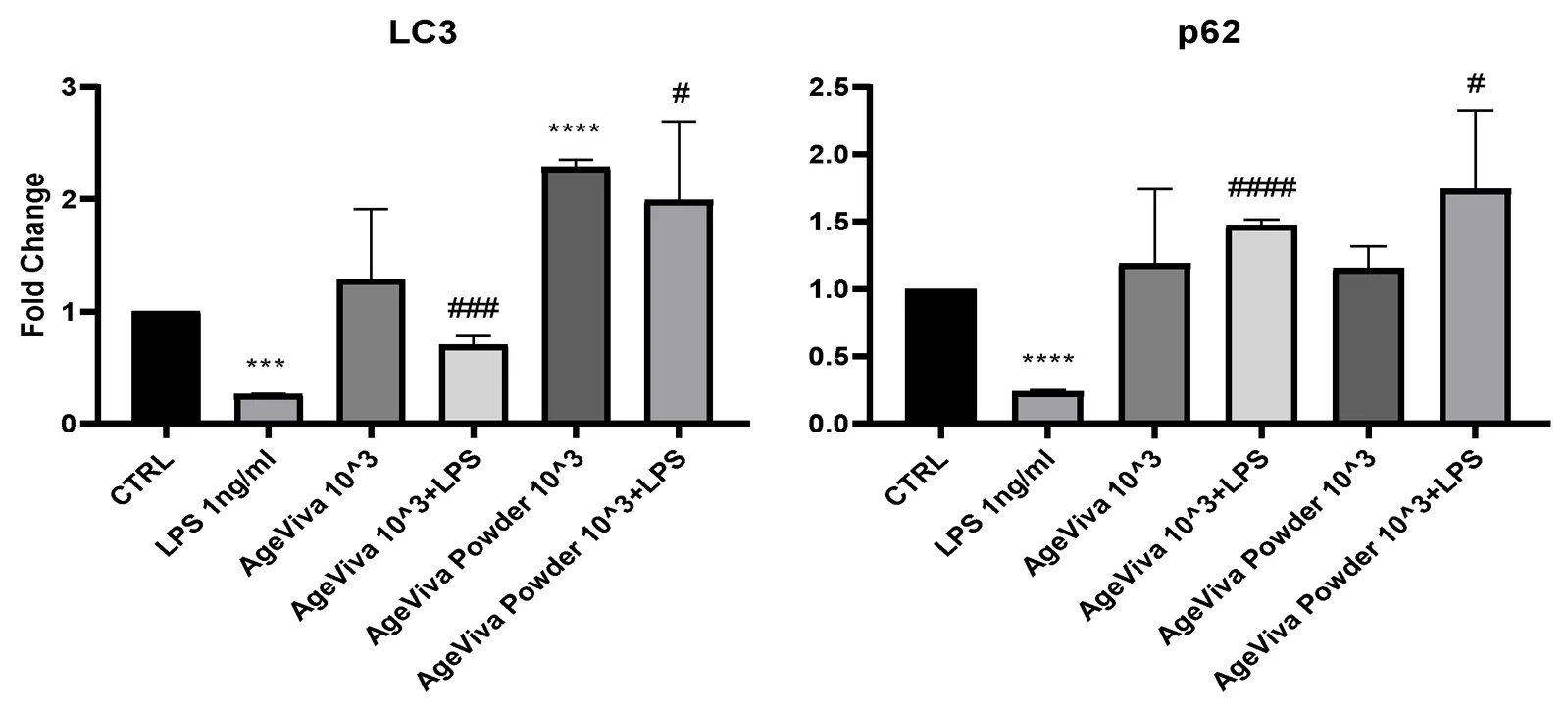
Analysis of gene expression (RT-qPCR) of the autophagic markers LC3 (**left**) and p62 (**right**) in BV2 cells. Data represent the mean fold change (FC) ± SEM. Statistical symbols: *** *p* < 0.001 and **** *p* < 0.0001 vs. CTRL; # *p* < 0.05 ### *p* < 0.001 and #### *p* < 0.0001 vs. LPS.

To investigate the role of AgeViva and AgeViva powder on autophagy processes, we analyzed the expression of LC3, involved in autophagosome formation, and Sequestrosome 1 (p62/SQSTM1) (a cargo protein degraded during autophagy) mRNAs (Figure 4). The induction of inflammatory stress via LPS (1 ng/mL) resulted in a significant downregulation of LC3 mRNA levels compared to the control (*p* < 0.001), suggesting a potential impairment of the autophagic machinery induced by the pro-inflammatory stimulus. Pre-treatment with AgeViva (10^3^) showed an increase in LC3 mRNA levels compared to LPS alone (*p* < 0.0001), bringing its expression back to levels close to the control. The AgeViva powder induced a marked increase in LC3 mRNA, both by itself (*p* < 0.0001) and as a pretreatment before LPS addition (*p* <0.05), suggesting a strong stimulation of autophagic gene transcription. Consistent with the LC3 data, treatment with LPS significantly reduced p62 expression compared to the control (*p* < 0.0001). Both pretreatments with AgeViva or AgeViva Powder counteracted the effect of LPS. A significant increase in p62 mRNA was detected in cells pretreated with AgeViva compared to cells treated with LPS alone (*p* < 0.0001). The pretreatment with AgeViva Powder also led to an increase in p62 mRNA levels compared to the levels detected in BV2 cells treated with LPS only (*p* < 0.05).

### 3.5. AgeViva Formulations Counteract LPS-Induced Suppression and Activate Nrf2 and Antioxidant Genes Expression

**Figure 5:**
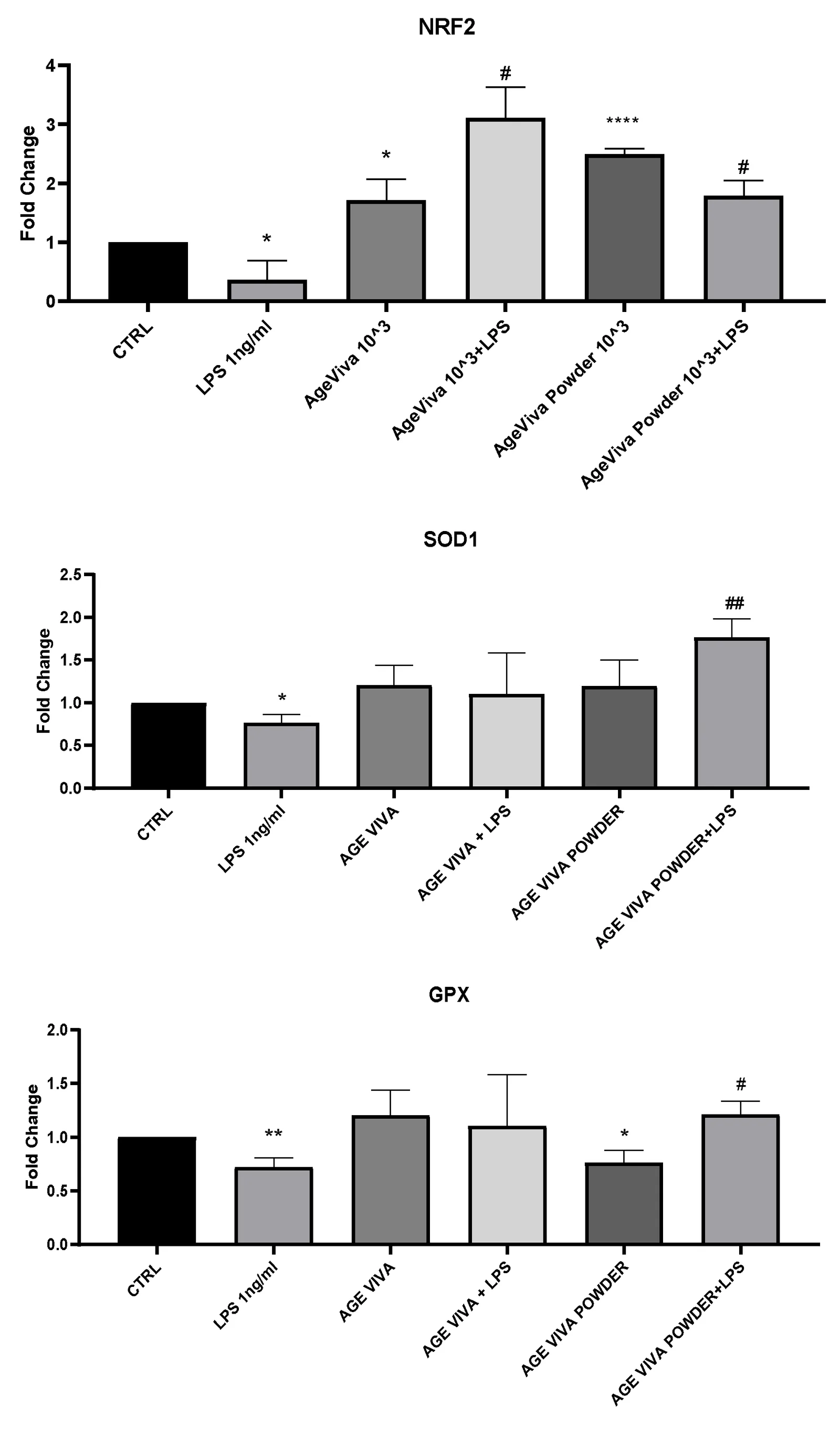
Analysis of gene expression (RT-qPCR) of Nrf2, SOD1 and GPX in BV2 cells. Data represent the mean FC ± SEM. Statistical symbols: * *p* < 0.05 ** *p* < 0.01 and **** *p* < 0.0001 vs. CTRL; # *p* < 0.05 ## *p* < 0.01 vs. LPS.

The bar graph illustrates the effects of LPS and two AgeViva formulations on Nrf2 expression, reported as fold change relative to the control (Figure 5). Treatment with LPS (1 ng/mL) markedly reduced Nrf2 expression compared with CTRL (#), indicating a strong inhibitory effect. In contrast, AgeViva (10^3^ dilution) increased Nrf2 (#). When combined with LPS, AgeViva (10^3^ + LPS) induced a pronounced upregulation of Nrf2 (*), representing the highest response observed in the experimental set. Similarly, AgeViva Powder (10^3^ dilution) elevated Nrf2 expression (####), while its combination with LPS resulted in a moderate increase (*), partially reversing the LPS-induced suppression. Gene expression of the antioxidant enzymes SOD1 and GPX was assessed in the different experimental groups and reported as a fold change relative to the control (Figure 5). In the group treated with LPS (1 ng/mL), a significant reduction in the expression of SOD1 (* *p* < 0.05 vs. CTRL) and GPX (** *p* < 0.01 vs. CTRL) was observed, indicating an alteration in the antioxidant response induced by the inflammatory stimulus. Treatment with AgeViva and AgeViva Powder showed a tendency to increase the expression of both genes compared to the LPS group, bringing levels back to or above those of the control. In particular, the AgeViva Powder + LPS combination resulted in the greatest increase in SOD1 expression, which was significantly higher than in the LPS group (## *p* < 0.01), and a significant increase in GPX as well (# *p* < 0.05 vs. LPS).

### 3.6. AgeViva Supplementation Improves Lifespan and Physiological Parameters in C. elegans

**Figure 6:**
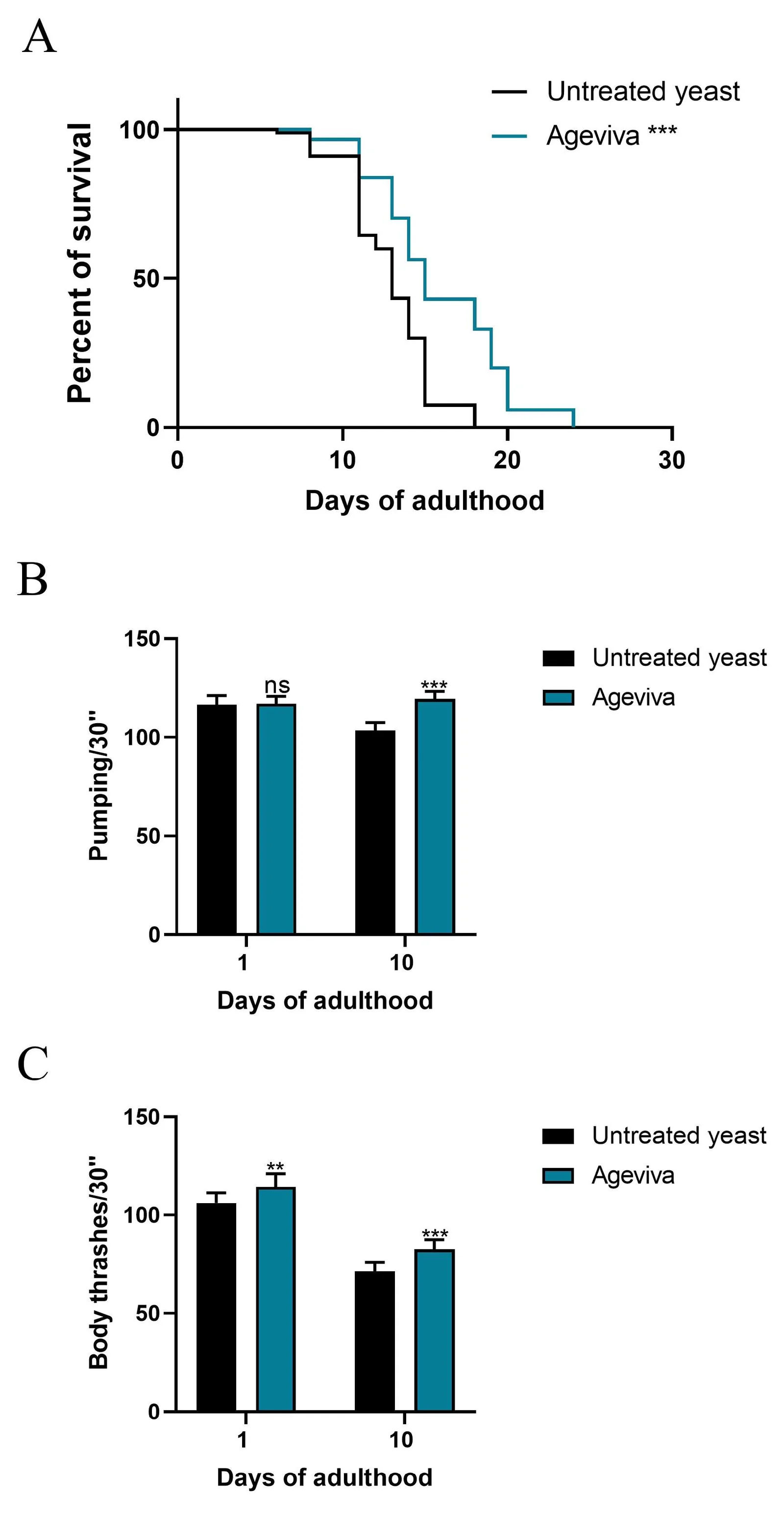
Effect of AgeViva supplementation on lifespan and healthspan parameters in *C. elegans*. (**A**) Kaplan–Meier survival curves of wild-type N2 nematodes fed with AgeViva yeast or untreated yeast from embryo hatching. *n* = 80 worms per condition in each independent experiment. (**B**) Pharyngeal pumping rate measured in 1-day-old and 10-day-old adult worms maintained on AgeViva or untreated yeast. Pumping activity was quantified over a 30 s interval under a stereomicroscope. *n* = 20 worms per condition in each independent experiment. (**C**) Locomotor activity assessed as body bending frequency in 1-day-old and 10-day-old adult nematodes fed untreated yeast or AgeViva yeast. Body bends were counted over a 30 s observation period. *n* = 20 worms per condition in each independent experiment. Statistical analysis was evaluated by two-way ANOVA with the Bonferroni post-test. Data are presented as mean ± SD from three independent experiments. Statistical significance is indicated as follows: ** *p* < 0.01; *** *p* < 0.001; ns: not significant.

To evaluate the effects of AgeViva supplementation on organismal physiology, lifespan analyses were performed in wild-type (WT) *C. elegans* maintained on AgeViva-supplemented plates from embryo hatching (Figure 6A). Worms fed with AgeViva exhibited a significant extension of median lifespan compared with control animals fed untreated yeast. In particular, 50% survival was reached at day 16 in the AgeViva-treated group compared to day 14 in the untreated yeast control, indicating a beneficial effect of the supplementation on longevity. In addition, the ageing markers pharyngeal pumping and body bending were analyzed to investigate whether AgeViva supplementation could influence feeding behaviour or locomotor activity in *C. elegans* during both early and adult stages. Pharyngeal pumping rate was evaluated as an indicator of food intake capacity in 1-day-old and 10-day-old adult worms (Figure 6B). At day 1 of adulthood, nematodes fed AgeViva displayed a similar pumping rate compared with untreated yeast-fed control. During ageing, a progressive decline in pumping activity was observed in control groups, as expected; however, at day 10 of adulthood, AgeViva-fed worms exhibited a significant increase in pumping activity of approximately 20% compared with control animals, suggesting that AgeViva supplementation preserves pharyngeal functionality during ageing and contributes to the maintenance of healthspan in *C. elegans*. Finally, locomotor activity, a well-established marker of healthspan that progressively declines during ageing, was assessed by measuring body bending frequency. As shown in Figure 6C, at day 1 of adulthood, worms fed AgeViva exhibited higher locomotor activity than untreated yeast-fed controls, with an increase of approximately 10% in body bending frequency. As expected, locomotor performance declined with ageing in the control group. However, on day 10 of adulthood, AgeViva-treated worms maintained significantly greater motility than control animals, displaying an approximately 20% higher body bending rate. These findings indicate that AgeViva supplementation attenuates the age-associated decline in locomotor function and contributes to the preservation of neuromuscular health during ageing.

### 3.7. AgeViva Preserves Muscle Mitochondrial Network Organization During Ageing

**Figure 7:**
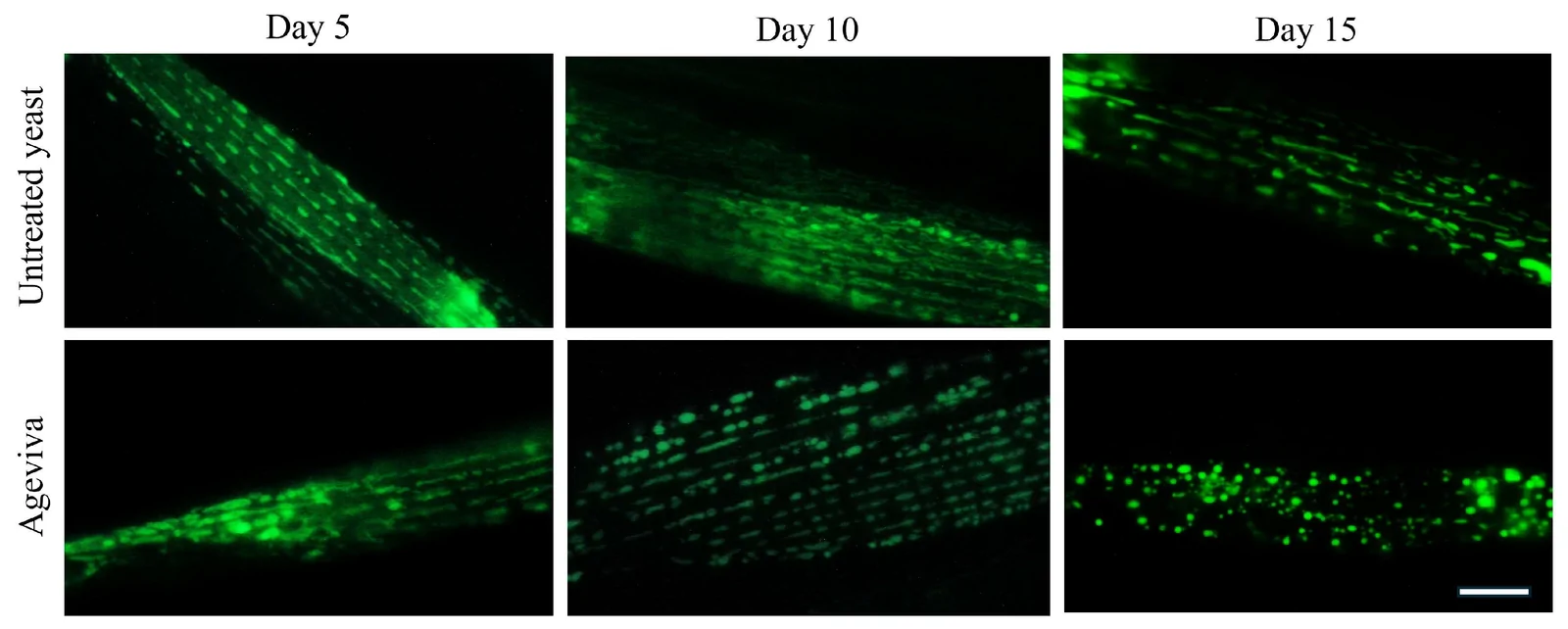
Effect of AgeViva supplementation on muscle integrity in *C. elegans*. Fluorescence microscopy of *myo-3*::GFP worms at 5, 10, and 15 days of adulthood treated with AgeViva. Worms fed untreated *S. cerevisiae* were taken as a control. *n* = 20 worms per condition in each independent experiment. Images are representative of three independent biological experiments. Scale bar: 5 μm.

Considering the central role of mitochondrial homeostasis in the regulation of ageing, neurodegenerative diseases, and oxidative stress, mitochondrial morphology was subsequently analyzed in *myo-3*::GFP worms. This strain expresses a mitochondrially targeted GFP under the control of the muscle-specific *myo-3* promoter, which drives fluorescence selectively in muscle tissue, allowing in vivo visualization of the mitochondrial network within muscle cells [14]. Since mitochondrial organization is closely associated with muscle homeostasis and cellular health, alterations in mitochondrial morphology can be used as an indicator of age-related muscle deterioration. During ageing, untreated yeast-fed worms showed a progressive alteration of the mitochondrial network, with a reduction in the continuity and organization of the mitochondrial network (Figure 7). In contrast, AgeViva-treated nematodes displayed distinct mitochondrial morphological features compared with untreated yeast-fed worms, including the presence of more rounded mitochondrial structures at later stages of ageing.

### 3.8. AgeViva Supplementation Reduces Cytosolic ROS Accumulation During Ageing

**Figure 8:**
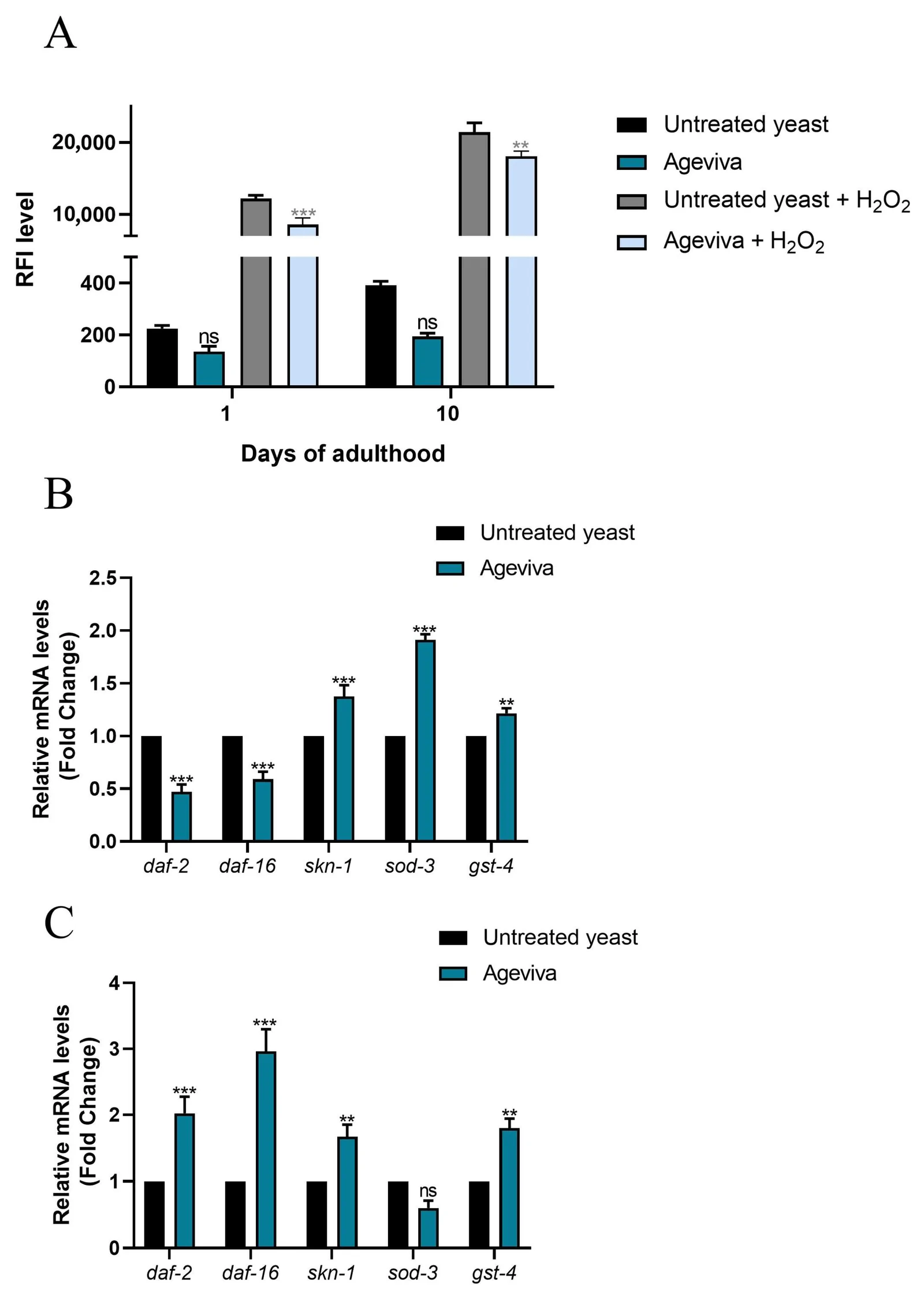
Analysis of oxidative stress in *C. elegans*. (**A**) Quantitative assessment of cytosolic ROS on day 1 and day 10 of adulthood. Values represent mean ± SD from three biological replicates (*n* = 50 worms/condition). (**B**) Expression levels of genes involved in oxidative stress responses (*daf-2*, *daf-16*, *skn-1*, *sod-3*, *gst-4*) analyzed by RT-qPCR in 1-day-old worms fed untreated yeast or AgeViva. Analyses were also performed in 10-day-old adult nematodes. n: 200 per condition (**C**). Gene expression levels were normalized to the corresponding control group. Histograms represent mean ± SD from three independent experiments performed in three independent experiments. Statistical significance was determined by two-way ANOVA followed by Bonferroni’s post-test. Asterisks indicate significant differences compared to untreated yeast-fed controls: ** *p* < 0.01; *** *p* < 0.001; ns: not significant.

Since oxidative imbalance is a hallmark of ageing, oxidative status was evaluated in AgeViva-supplemented worms under basal conditions and following hydrogen peroxide (H_2_O_2_) exposure as an external oxidative challenge (Figure 8A). On day 1 of adulthood, nematodes fed untreated yeast or AgeViva showed no significant difference in ROS accumulation under basal conditions, but exhibited reduced ROS fluorescence, of about 10%, in the presence of H_2_O_2_. During ageing, 10-day-old adult untreated yeast-fed worms displayed increased fluorescence levels, consistent with an age-associated increase in oxidative stress, while AgeViva-fed worms showed a significant 50% reduction in fluorescence under basal conditions. Lastly, even under external oxidative stress, worms fed AgeViva showed a 10% reduction in fluorescence levels. These results indicate that the AgeViva diet is associated with reduced oxidative stress-related fluorescence signals during ageing. In parallel, expression of *daf-16*, *daf-2*, *sod-3*, *gst-4*, and *skn-1* genes, associated with oxidative stress resistance and longevity pathways, was evaluated. The Insulin/IGF-1 signalling components *daf-2* and *daf-16* represent major regulators of ageing and stress adaptation in *C. elegans*, with *daf-16*/FOXO activation promoting stress resistance and lifespan extension [13]. *sod-3*, a downstream target of *daf-16*, encodes a mitochondrial SOD involved in detoxification of reactive oxygen species. *gst-4* is a phase II detoxification enzyme commonly used as a marker of oxidative stress responses, while *skn-1*, the ortholog of mammalian Nrf2 [15], controls antioxidant and cytoprotective pathways involved in cellular defence against oxidative damage [16]. At day 1 of adulthood (Figure 8B), *skn-1* expression increased by approximately 30%, while *sod-3* transcript levels showed a two-fold increase and *gst-4* expression was upregulated by approximately 25% in AgeViva-treated worms compared with untreated yeast-fed controls. In contrast, both *daf-2* and *daf-16* transcript levels were reduced by approximately 50%. Collectively, these findings suggest that AgeViva promotes an early activation of antioxidant defence mechanisms, likely through the induction of *skn-1*-dependent stress-response pathways, independently of an early activation of the canonical IIS signalling cascade. By day 10 of adulthood (Figure 8C), *daf-2* and *daf-16* transcript levels were both approximately two-fold higher in AgeViva-treated worms than in controls. In addition, *skn-1* and *gst-4* expression increased by nearly 50%, whereas *sod-3* expression was not significantly different between the two groups. Given the central role of the *daf-2*/*daf-16* signaling axis in the regulation of stress resistance and longevity, together with the sustained upregulation of *skn-1* and *gst-4*, these findings suggest that AgeViva enhances adaptive responses to oxidative stress and contributes to the maintenance of cellular homeostasis during ageing.

### 3.9. AgeViva Attenuates Age-Associated Mitochondrial Superoxide Accumulation

**Figure 9:**
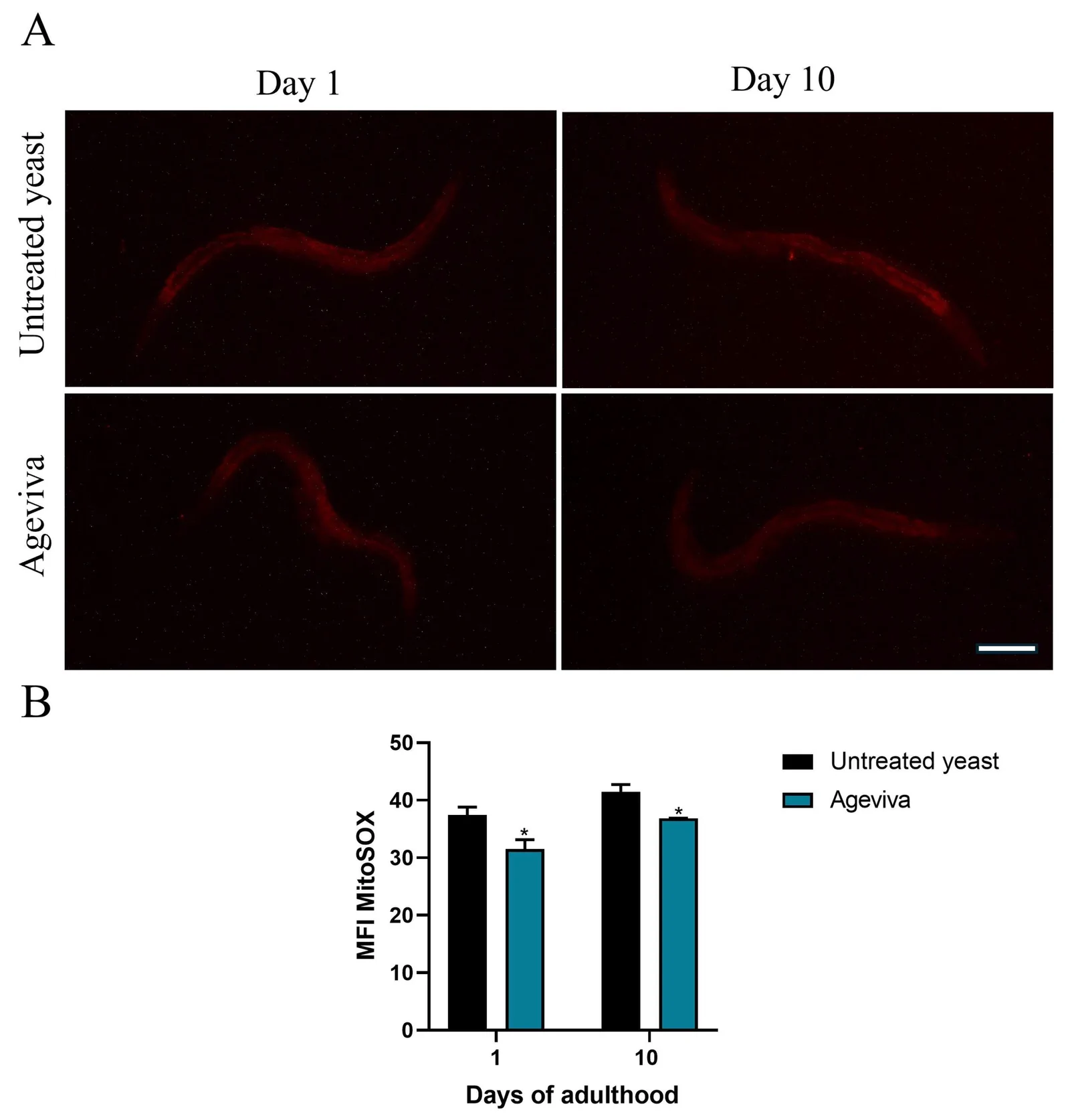
Mitochondrial ROS levels in *C. elegans* grown on AgeViva. (**A**) Representative fluorescence images of 1-day-old and 10-day-old adult worms fed AgeViva or untreated yeast (control) and stained with MitoSOX™ Red to assess mitochondrial superoxide production during ageing. *n* = 20 worms per condition in each independent experiment. Images are representative of three independent biological experiments. Scale bar: 100 μm. (**B**) Quantification of MitoSOX™ Red fluorescence expressed as mean fluorescence intensity (MFI) in the different experimental groups. Statistical analysis was evaluated by two-way ANOVA with the Bonferroni post-test; data are presented as mean ± SD from three independent experiments. Statistical significance is indicated as follows: * *p* < 0.05.

To better characterize the oxidative status of mitochondria, mitochondrial superoxide generation was subsequently analyzed using MitoSOX fluorescence staining. Indeed, since excessive mitochondrial ROS accumulation is a hallmark of ageing and mitochondrial dysfunction, changes in MitoSOX fluorescence can be used as an indicator of oxidative stress levels in vivo [17]. As illustrated in Figure 9, MitoSOX fluorescence analysis revealed that mitochondrial superoxide levels were already significantly reduced in AgeViva-treated worms at day 1 of adulthood. Quantitative analysis of mean fluorescence intensity (MFI) showed an approximately 25% decrease in mitochondrial ROS production compared with untreated yeast-fed controls (Figure 9B). As expected, mitochondrial oxidative stress increased with ageing in control worms, as evidenced by the marked increase in MitoSOX fluorescence observed at day 10. In contrast, AgeViva-treated nematodes exhibited significantly lower mitochondrial superoxide levels, with MitoSOX fluorescence intensity reduced by approximately 20% compared with aged untreated yeast-fed controls. These findings indicate that AgeViva supplementation attenuates mitochondrial superoxide accumulation throughout adulthood, contributing to the maintenance of mitochondrial redox homeostasis during ageing.

### 3.10. Maintenance of Mitochondrial Function and Homeostasis by AgeViva Supplementation

**Figure 10:**
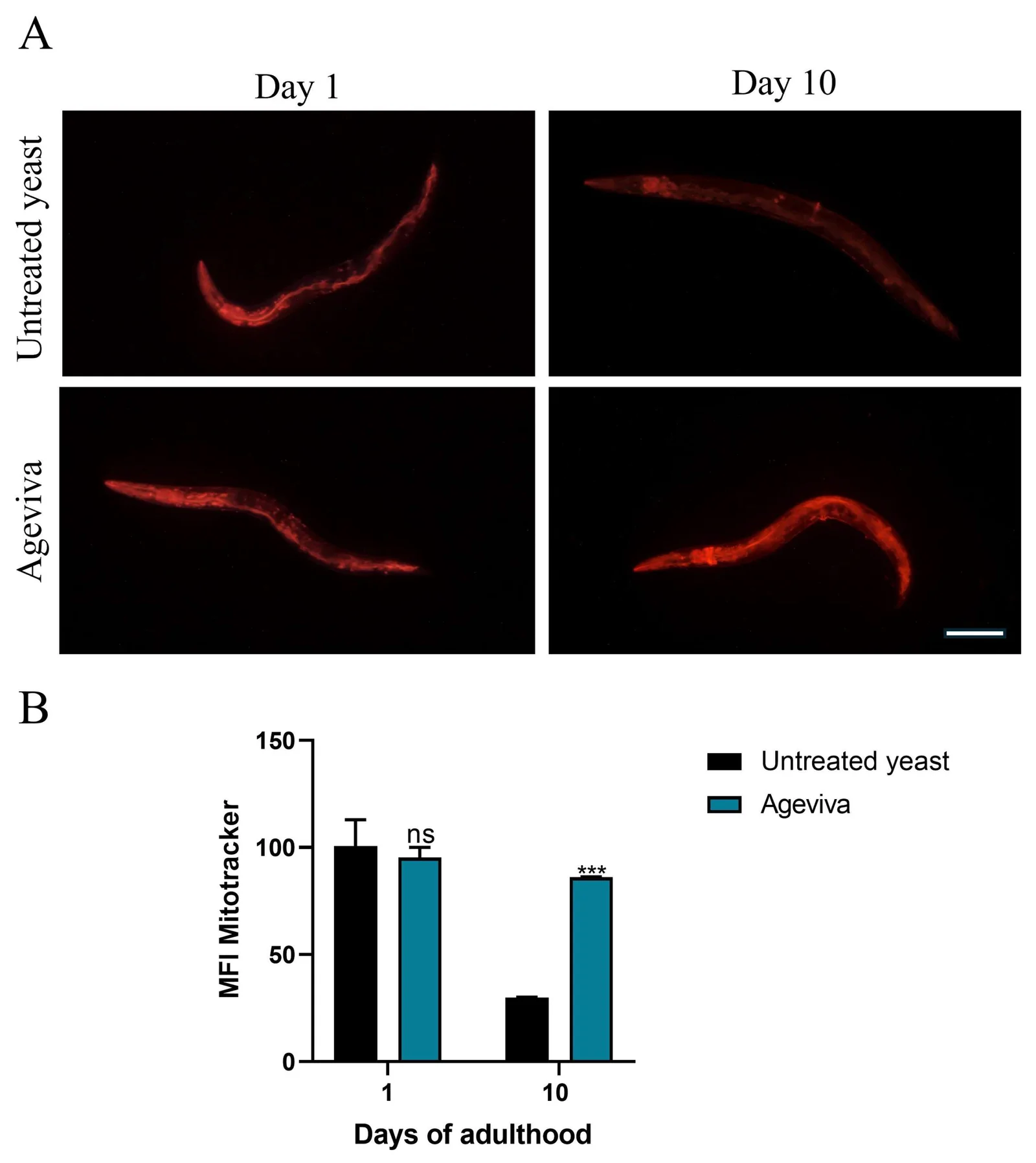
Effect of AgeViva supplementation on mitochondrial-associated fluorescence during ageing in *C. elegans*. (**A**) Representative fluorescence images of 1- and 10-day-old adult nematodes fed AgeViva or untreated yeast and stained with MitoTracker^®^ Red CMXRos to evaluate mitochondrial-associated fluorescence during ageing. *n* = 20 worms per condition in each independent experiment. Images are representative of three independent biological experiments. Scale bar: 100 μm. (**B**) Quantification of mitochondrial fluorescence expressed as MFI in the different experimental groups. Statistical analysis was evaluated by two-way ANOVA with the Bonferroni post-test; data are presented as mean ± SD from three independent experiments. Statistical significance is indicated as follows: *** *p* < 0.001, ns: not significant.

To further characterize age-associated changes in mitochondrial fluorescence, MitoTracker^®^ Red CMXRos staining was evaluated in AgeViva- and untreated yeast-fed worms. MitoTracker^®^ Red CMXRos accumulates in mitochondria of live cells, and its fluorescence signal is influenced by multiple factors, including mitochondrial membrane potential and mitochondrial content. As shown in Figure 10A, ageing untreated yeast-fed worms exhibited a progressive reduction in MitoTracker fluorescence. In contrast, AgeViva-treated nematodes displayed higher and more homogeneous mitochondrial-associated fluorescence throughout ageing. Quantitative analysis of mean fluorescence intensity (MFI) supported these observations (Figure 10B). No significant differences were detected between the experimental groups at day 1 of adulthood. However, by day 10 of adulthood, AgeViva-treated worms displayed a 2-fold increase in MitoTracker fluorescence compared with untreated yeast-fed controls (Figure 10B), indicating higher mitochondrial-associated fluorescence at this time point [18].

The observed preservation of mitochondrial function, together with the reduction in mitochondrial ROS accumulation in AgeViva-treated worms, suggested the involvement of cellular quality-control mechanisms responsible for maintaining mitochondrial homeostasis. Given the well-established interplay between autophagy, mitochondrial turnover, and oxidative stress during ageing [19,20], we next investigated the expression of genes involved in autophagic pathways and stress-response signalling.

**Figure 11:**
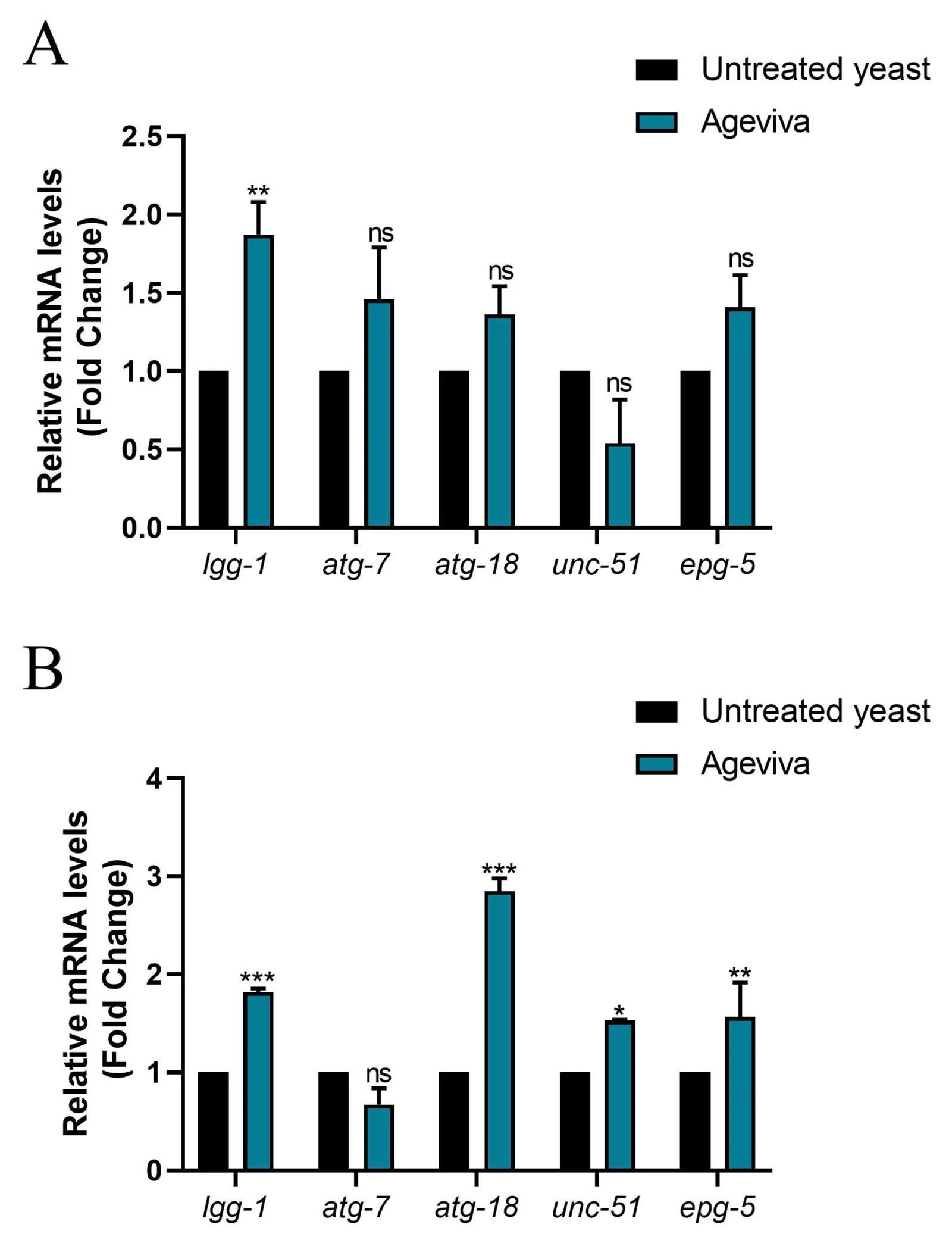
Impact of AgeViva on autophagy pathways in *C. elegans*. Expression levels of genes involved in autophagy regulation (*lgg-1*, *atg-7*, *atg-18*, *unc-51*, *epg-5*) in wild-type N2 worms fed untreated yeast or AgeViva. Analyses were performed in (**A**) 1-day-old and (**B**) 10-day-old adult nematodes. Gene expression levels were quantified by real-time PCR and normalized to the corresponding control group (n: 200 in each condition). Histograms represent mean ± SD from three independent experiments performed in triplicate. Statistical significance was determined by two-way ANOVA followed by Bonferroni’s post-test. Asterisks indicate significant differences compared to untreated yeast-fed controls: * *p* < 0.05; ** *p* < 0.01; *** *p* < 0.001, ns: not significant.

RT-qPCR analysis was performed in 1-day-old and 10-day-old adult *C. elegans* to investigate the effects of AgeViva supplementation on the expression of genes involved in autophagy during ageing. In particular, the transcriptional levels of key autophagy-related genes, including *lgg-1*, *atg-7*, *atg-18*, *unc-51*, and *epg-5*, were analyzed. *lgg-1* encodes the *C. elegans* ortholog of mammalian LC3 and represents a central marker of autophagosome formation [21]. *atg-7* and *atg-18* are essential components of the autophagic machinery required for autophagosome biogenesis and membrane expansion, whereas unc-51, the ortholog of mammalian Autophagy Activating Kinase 1 (ULK1), regulates the initiation of autophagy. *epg-5* is involved in autophagosome maturation and lysosomal fusion, playing a critical role in autophagic flux completion [21]. At day 1 of adulthood (Figure 11A), AgeViva supplementation selectively modulated the expression of autophagy-related genes. Specifically, *lgg-1* transcript levels significantly increased by approximately 50% compared with untreated yeast-fed controls, whereas the expression of *atg-7*, *atg-18*, *epg-5*, and *unc-51* did not differ significantly between the two groups. These findings suggest an early and selective induction of autophagy, primarily associated with the upregulation of *lgg-1*. A distinct transcriptional profile was observed in aged worms (Figure 11B). At day 10 of adulthood, *lgg-1* expression remained significantly elevated (approximately 50%), while *atg-18* exhibited a 2.5-fold increase and *epg-5* was upregulated by approximately 45% compared with untreated yeast-fed controls. In contrast, *atg-7* and *unc-51* transcript levels were not significantly different between the two groups. The sustained upregulation of genes involved in autophagosome formation and maturation suggests that AgeViva contributes to the maintenance of autophagic processes during ageing, potentially supporting cellular homeostasis and longevity.

## 4. Discussion

The present study demonstrates that AgeViva supplementation exerts widespread protective effects on both cellular and organismal ageing models by modulating inflammatory responses, oxidative stress, autophagy, and mitochondrial homeostasis. Using BV2 microglial cells and *C. elegans* as complementary experimental systems, we provide evidence that AgeViva promotes a cellular environment associated with enhanced stress resistance and improved physiological function during ageing. The addition of AgeViva to LPS-stimulated BV2 cells showed a reduction in the production of inflammatory mediators as well as an increase in anti-inflammatory markers, demonstrating an AgeViva-driven anti-inflammatory polarization of glial cells. It also demonstrated the restoration of autophagic mechanisms impaired by the addition of LPS. AgeViva was also shown to act on redox balances by decreasing the production of superoxide anion and promoting the expression of the transcription factor Nrf2, which activates the transcription of antioxidant enzymes. Moreover, the data from the cellular model suggest that AgeViva adjusts mitochondrial function, redox homeostasis, and autophagy. A bidirectional interaction between immune activation and cellular metabolism, known as immunometabolism, bridging immune signalling and metabolism, has been recently recognized as a key modulator of chronic inflammation, outlining the central role of mitochondria in the development of chronic diseases impacting longevity. Consistent with the in vitro findings, AgeViva supplementation improved both lifespan and healthspan parameters in *C. elegans*. Importantly, AgeViva-treated worms maintained higher pharyngeal pumping and locomotor activity throughout adulthood. Since the progressive decline in feeding behaviour and movement is a well-established hallmark of ageing in *C. elegans* [22], the preservation of muscular and physiological functions suggests that AgeViva promotes not only increased survival but also a healthier ageing phenotype. The beneficial effects of AgeViva were associated with the modulation of key molecular pathways involved in cellular quality control and stress adaptation. In particular, AgeViva supplementation influenced the expression of several genes involved in autophagy, including *lgg-1*, *atg-7*, *atg-18*, *unc-51*, and *epg-5*, suggesting a modulation of pathways regulating autophagosome formation, maturation, and turnover. Autophagy plays a central role in the maintenance of cellular homeostasis and has been extensively linked to lifespan regulation in *C. elegans* and other model organisms [23]. During ageing, a progressive decline in autophagic efficiency contributes to the accumulation of damaged proteins and dysfunctional organelles, ultimately promoting cellular dysfunction [24]. Conversely, enhanced autophagic activity has been associated with improved stress resistance and lifespan extension in several longevity paradigms. Therefore, the transcriptional modulation observed in AgeViva-treated worms may represent one of the mechanisms underlying the improved physiological performance and longevity observed in this study. In parallel, AgeViva modulated the expression of genes involved in oxidative stress responses and insulin/IGF-1 signalling pathways, including *daf-2*, *daf-16*, *skn-1*, *sod-3*, and *gst-4*. These pathways are among the most important regulators of ageing and stress adaptation in *C. elegans* [25]. In particular, DAF-16/FOXO and *skn-1*/Nrf2 function as master transcriptional regulators of antioxidant, detoxification, and cytoprotective responses, promoting organismal resilience under stress conditions and contributing to lifespan extension. The observed modulation of these genes, based on transcriptional analysis, is consistent with the reduced accumulation of cytosolic and mitochondrial oxidative stress detected in AgeViva-treated worms. Interestingly, previous studies demonstrated that vitamin D3 promotes longevity and improves proteostasis in *C. elegans* through the modulation of stress-response pathways involving *skn-1*, Inositol-requiring enzyme 1 (*ire-1*), and X-box binding protein 1 (*xbp-1*) genes [26]. Similarly, zinc homeostasis has been directly linked to lifespan regulation through *daf-16*, Heat Shock Factor 1 (HSF-1), and SKN-1-dependent mechanisms, highlighting the critical role of redox balance in ageing processes [27]. Mitochondrial homeostasis emerged as another major target of AgeViva supplementation. Ageing untreated yeast-fed worms displayed the expected deterioration of the mitochondrial network in body-wall muscle cells, characterized by progressive fragmentation and loss of structural organization, a phenotype commonly associated with sarcopenia and mitochondrial dysfunction [28]. In contrast, AgeViva-treated animals preserved a more organized mitochondrial architecture throughout ageing. This structural preservation was accompanied by sustained MitoTracker fluorescence and reduced MitoSOX signal in aged worms, indicating improved maintenance of mitochondrial membrane potential and lower mitochondrial superoxide accumulation. Given the central role of mitochondria as both a source and target of reactive oxygen species, these findings suggest that AgeViva contributes to the preservation of mitochondrial functionality and redox homeostasis during ageing. The protective effects observed in *C. elegans* are likely attributable to the combined action of the bioactive components present in the formulation. Zinc is an essential cofactor for numerous antioxidant enzymes and plays a crucial role in cellular stress responses [29], whereas vitamin D3 has been associated with anti-inflammatory, cytoprotective, and longevity-promoting pathways [26,30]. In addition, pantothenic acid, as a precursor of coenzyme A, is fundamental for mitochondrial metabolism and energy production [31]. Notably, recent evidence has identified pantothenic acid as a metabolite capable of preserving mitochondrial integrity and delaying muscle ageing in *C. elegans* [32]. Collectively, these observations support the hypothesis that the combination of zinc, vitamin D3, and pantothenic acid within the AgeViva formulation appears to exert potentially additive effects on autophagy, oxidative stress resistance, and mitochondrial homeostasis, thereby contributing to the extension of lifespan and the preservation of healthspan observed in the present study. Particular interest has arisen from the results pertaining to the functional state of mitochondria. As a matter of some magnitude, mitochondria have been recognized increasingly as drivers of ageing, bioenergetics, redox control and innate immunity [33]. Mitochondrial dysfunction leads to misfolded proteins, defective clearance, alteration in metabolite availability, induction of ROS overproduction, and NFkB and cyclic GMP-AMP synthase (cGAS-STING) pathways that reinforce chronic inflammatory tone [34,35,36]. Alterations in mitochondrial function are observed in ageing, in which there is an increasing electron leakage from the electron transport chain, with increased oxidative stress and consequent damage to lipids, proteins, and DNA. Disruption of metabolic-immune homeostasis may underlie the susceptibility of the ageing brain to neurodegeneration [37]. Although our data demonstrate that AgeViva preserves muscle mitochondrial morphology and reduces mitochondrial superoxide levels, these findings should not be over-interpreted as direct evidence of neuroprotection, gut microbiota regulation, or therapeutic efficacy in clinical neurodegeneration. Importantly, the recent literature highlights the intricate interplay between gut microbiota dynamics, mitochondrial redox dysfunction, and systemic metabolic homeostasis, emphasizing the necessity of distinguishing conceptual intervention frameworks from direct mechanistic evidence [38]. Because our study did not measure gut microbial composition, microbial-derived metabolites, neuronal survival, or clinical endpoints, our findings should be viewed strictly as hypothesis-generating. Future investigations utilizing genetic pathway validation, formulation component deconvolution, and disease-relevant mammalian models will be necessary to determine whether the cellular and mitochondrial responses observed here can be translated into targeted interventions for age-associated pathophysiology [38]. It is important to emphasize how the complex matrix of AgeViva provides a distinct functional profile compared to single-ingredient Milmed yeast reported previously [5]. While our prior work established the baseline involvement of the SKN-1/Nrf2 pathway and mTOR-dependent autophagy, the current findings reveal that co-formulation with micronutrients specifically reinforces mitochondrial homeostasis—as evidenced by the preservation of body-wall muscle mitochondrial architecture (myo-3::GFP) and reduced mitochondrial ROS generation (MitoSOX). Furthermore, the demonstrated expression profile of anti-inflammatory/M2 polarization-associated markers (Arginase-1 vs. iNOS) in BV-2 cells underscores a novel immunomodulatory capacity of the AgeViva complex that extends beyond general antioxidant defence.

These findings are consistent with previous experimental studies demonstrating that natural bioactive compounds can attenuate oxidative stress by reducing oxidative damage and/or enhancing endogenous antioxidant defence mechanisms. Natural bioactive compounds (such as flavonoids, stilbenes, terpenes, alkaloids, saponins, polysaccharides, etc.) have been reported to exhibit a multitude of health-promoting effects, including antioxidant activity [38]. In particular, naturally occurring bioactive compounds such as sulforaphane, curcumin, and resveratrol were shown to attenuate oxidative stress and inflammatory signalling [39].

Our previous results demonstrated the antioxidant and anti-inflammatory properties of tomato-derived bioactives [40], as well as the activity of curcumin counteracting oxidative stress and neuroinflammation [41] and the anti-inflammatory and antioxidant activity of wheat seeds, flour, chaff, and pasta [42].

Other relevant and recent experimental studies outlined the protective effects of natural bioactive compounds against oxidative damage [43].

Moreover, natural compounds, such as polyphenols present in several fruit and vegetable species, have been shown to counteract imbalances related to mitochondrial dysfunction. The implementation of new treatments targeting non-communicable diseases that develop mainly in the elderly is aimed at stimulating mitogenesis, correct mitochondrial metabolic reprogramming, and autophagic machinery to eliminate aged or no longer functional mitochondria. Our results seem to fully converge upon this aim.

## 5. Conclusions

In conclusion, the present study provides preliminary and hypothesis-generating evidence that AgeViva supplementation may influence cellular and organismal parameters related to inflammatory responses, oxidative stress, autophagy, and mitochondrial homeostasis in BV-2 microglial cells and *C. elegans*. Under the experimental conditions investigated, AgeViva treatment was associated with reduced expression of pro-inflammatory transcripts, increased expression of antioxidant-related markers, preservation of skeletal muscle mitochondrial structural features, and improvements in basal healthspan-related parameters, including locomotor activity and pharyngeal pumping. Nevertheless, these findings should be interpreted within the specific experimental framework of the study, and several limitations need to be considered. In particular, the current work does not provide direct evidence of neuroprotective effects, as it did not include primary neuronal cultures, neuron–microglia co-culture systems, or disease-relevant neurodegenerative models. Therefore, while the observed modulation of inflammatory, oxidative stress, and mitochondrial-related pathways supports the potential biological activity of AgeViva, further studies using more physiologically relevant neuronal models and disease contexts will be required to determine whether these effects translate into functional neuroprotection.

## Figures and Tables

**Table 1:** List of primers.

GENE	Forward Primer (5′–3′)	Reverse Primer (5′–3′)	Accession Numbers
*mLC3*	TTCTTCCTCCTGGTGAATGG	GTCTCCTGCGAGGCATAAAC	NM_026160
*mNrf2*	TCTGAGCCAGGACTACGACG	GAGGTGGTGGTGGTGTCTCTGC	NM_031789
*mp62*	CCTTGCCCTACAGCTGAGTC	CCACACTCTCCCCCACATTC	NM_001290769
*mIL-1β*	GAAATGCCACCTTTTGACAGTG	TGGATGCTCTCATCAGGACAG	NM_008361.4
*mIL-10*	GCCCTTTGCTATGGTGTCCTTTC	TCCCTGGTTTCTCTTCCCAAGAC	NM_010548.2
*mTNF-α*	CTGAACTTCGGGGTGATCGG	GGCTTGTCACTCGAATTTTGAGA	BC137720.1
*mARG1*	ATGTGCCCTCTGTCTTTTAGGG	GGTCTCTCACGTCATACTCTGT	NM_007482.3
*miNOS*	GGCAGCCTGTGAGACCTTTG	GCATTGGAAGTGAAGCGTTTC	AF427516.1
*mβ-Actin*	GGCTGTATTCCCCTCCATCG	CCAGTTGGTAACAATGCCATGT	NM_007393.5
*mSOD1*	GCCCGCTAAGTGCTGAGTC	AGCCCCAGAAGGATAACGGA	NM_017050
*mGPX*	CCACCGTGTATGCCTTCTCC	AGAGAGACGCGACATTCTCAAT	NM_008160

**Table 2:** Primers used for real-time qPCR.

GENE	Forward Primer (5′–3′)	Reverse Primer (5′–3′)
*act-1*	5′-GAGCGTGGTTACTCTTTCA	5′-CAGAGCTTCTCCTTGATGTC
*atg-7*	5′-CCAAAAGCTGTGGGATGGGA	5′-GCGTTCCAGCACCAAGAATG
*atg-18*	5′-CAGGAGCCGCAAGGAGTAAT	5′-CGATTGGTTGCTTGCTTCGG
*daf-2*	5′-ATCGTCGGATTCTACTGTACTCCC	5′-CCGACATCTGACAATATTCATTCTCGT
*daf-16*	5′- TCAAGACCTCAAAGCCAATCAACTC	5′-ACGAGAAAGAAGGAGTAAGAGGA
*epg-5*	5′-CTCCACCACCACGTGTTTC	5′-TGGTGCTACCGCTGTAGTTG
*gst-4*	5′-TCAATGTGCCTTACGAGGATTA	5′-CGAATTGTTCTCCATCGACTTG
*lgg-1*	5′-GCCGAAGGAGACAAGATCCG	5′-GGTCCTGGTAGAGTTGTCCC
*pmp-3*	5′-GTTCCCGTGTTCATCACTCAT	5′-ACACCGTCGAGAAGCTGTAGA
*skn-1*	5′-AGTGTCGGCGTTCCAGATT	5′-GTCGACGAATCTTGCGAAT
*sod-3*	5′-AGAACCTTCAAAGGAGCTGATG	5′-CCGCAATAGTGATGTCAGAAAG
*unc-51*	5′-CGCCGGTGGTTCAGCGGATT	5′-TATCCTGGGTGTCGGCGGGG

## Data Availability

The data presented in this study are available from the corresponding author upon reasonable request.

## Abbreviations

LPS	Lipopolysaccharide
*C. elegans*	*Caenorhabditis elegans*
ROS	Reactive Oxygen Species
IL-1Β	Interleukin-1 Beta
TNF-A	Tumor Necrosis Factor Alpha
IL-10	Interleukin-10
LC3	Microtubule-Associated Protein 1a/1b-Light Chain
p62	Sequestosome 1
Nrf2	Nuclear Factor Erythroid 2-Related Factor 2
SOD1	Superoxide Dismutase 1
GPX	Glutathione Peroxidase
INSULIN/IGF-1	Insulin-like Growth Factor 1
DAF-2	Dauer Formation-2
DAF-16	Dauer Formation-16
SKN-1	Skinhead-1
LGG-1	Ligating
ATG-7	Autophagy-Related 7
ATG-18	Autophagy-Related 18
UNC-51	Uncoordinated 51
EPG-5	Ectopic P-Granules Autophagy Protein 5
AD	Alzheimer’s Disease
PD	Parkinson’s Disease
ALS	Amyotrophic Lateral Sclerosis
IL-6	Interleukin-6
NF-ΚB	Nuclear Factor Kappa-Light-Chain-Enhancer Of Activated B Cells
DNA	Deoxyribonucleic Acid
O_2_^−^	Superoxide Anion
H_2_O_2_	Hydrogen Peroxide
OH	Hydroxyl Radical
EDTA	Ethylenediaminetetraacetic Acid
RNA	Ribonucleic Acid
CDNA	Complementary Dna
RT-qPCR	Real-Time Quantitative Polymerase Chain Reaction
CT	Threshold Cycle
Δ CT	Cycle Threshold Difference
NGM	Nematode Growth Medium
YPD	Yeast Extract Peptone Dextrose
MYO-3	Myosin-3
GFP	Green Fluorescent Protein
H2DCF-DA	2′,7′-Dichlorodihydrofluorescein Diacetate
SD	Standard Deviation
iNOS	Inducible Nitric Oxide Synthase
mRNA	Messenger Ribonucleic Acid
ARG-1	Arginase-1
CTRL	Control
p62/SQSTM1	Sequestosome 1
FC	Fold Change
SEM	Standard Error of the Mean
WT	WILD TYPE
SOD 3	Superoxide Dismutase 3
MFI	Mean Fluorescence Intensity
ULK1	Autophagy Activating Kinase 1
ire-1	Inositol-requiring enzyme 1
xbp-1	X-box binding protein 1
HSF-1	Heat Shock Factor 1
cGAS-STING	Cyclic GMP-AMP Synthase
